# Impairment of Meristem Proliferation in Plants Lacking the Mitochondrial Protease AtFTSH4

**DOI:** 10.3390/ijms19030853

**Published:** 2018-03-14

**Authors:** Alicja Dolzblasz, Edyta M. Gola, Katarzyna Sokołowska, Elwira Smakowska-Luzan, Adriana Twardawska, Hanna Janska

**Affiliations:** 1 Faculty of Biological Sciences, Institute of Experimental Biology, Kanonia 6/8, 50-328 Wroclaw, Poland; edyta.gola@uwr.edu.pl (E.M.G.); katarzyna.sokolowska@uwr.edu.pl (K.S.); adriana.twardawska@uwr.edu.pl (A.T.); 2 Faculty of Biotechnology, University of Wroclaw, F. Joliot-Curie 14A, 50-383 Wroclaw, Poland; elwira.smakowska@gmi.oeaw.ac.at (E.S.-L.); hanna.janska@uwr.edu.pl (H.J.)

**Keywords:** Arabidopsis, cell divisions, mitochondria, oxidative stress, root apical meristem, shoot apical meristem

## Abstract

Shoot and root apical meristems (SAM and RAM, respectively) are crucial to provide cells for growth and organogenesis and therefore need to be maintained throughout the life of a plant. However, plants lacking the mitochondrial protease AtFTSH4 exhibit an intriguing phenotype of precocious cessation of growth at both the shoot and root apices when grown at elevated temperatures. This is due to the accumulation of internal oxidative stress and progressive mitochondria dysfunction. To explore the impacts of the internal oxidative stress on SAM and RAM functioning, we study the expression of selected meristem-specific (*STM*, *CLV3*, *WOX5*) and cell cycle-related (e.g., *CYCB1*, *CYCD3;1*) genes at the level of the promoter activity and/or transcript abundance in wild-type and loss-of-function *ftsh4-1* mutant plants grown at 30 °C. In addition, we monitor cell cycle progression directly in apical meristems and analyze the responsiveness of SAM and RAM to plant hormones. We show that growth arrest in the *ftsh4-1* mutant is caused by cell cycle dysregulation in addition to the loss of stem cell identity. Both the SAM and RAM gradually lose their proliferative activity, but with different timing relative to *CYCB1* transcriptional activity (a marker of G2-M transition), which cannot be compensated by exogenous hormones.

## 1. Introduction

Plant growth and development are enabled by the activity of the shoot and root apical meristems (SAM and RAM, respectively). The continuous maintenance of stem cells in the SAM and RAM is facilitated by signaling from the organizing center (OC) and quiescent center (QC), respectively [1,2]. The main roles of the SAM and RAM are analogous, but both meristems differ in terms of internal organization and localization of growth and organogenic activity [3]. Fundamental to SAM/RAM self-perpetuation are sustained cell divisions, as the meristem shape and size need to be maintained while continuously providing cells for development. Consequently, plant growth strongly depends on precisely coordinated cell proliferation and differentiation within various subdomains of the SAM and RAM [4,5,6,7]. Meristem regulators must be accurately interpreted by the cell cycle machinery, which in turn feedbacks on the production of meristem regulators (e.g., [2]). In addition, cell cycle perturbations impair the response of the meristem to extrinsic signals including hormones and metabolic sugars [8,9,10,11].

Cell cycle regulation in higher plants depends primarily on cyclins (CYCs) and their interacting partners including several cyclin-dependent kinases (CDKs) [12]. *CDKA1* is a major cell-cycle controlling CDK in *Arabidopsis*, needed for both G1-S and G2-M transitions [13,14]. The most extensively studied cell cycle control proteins in reference to proper SAM functioning are the regulators of G1-S transition, *CYCD3s*, which extend the mitotic window of the cells [15,16], and the regulators of G2-M transition, *CYCB1;1* and *CDKB2* [8,16]. Surprisingly, the modification of cell division rates does not often result in pronounced architectural changes in the plant body [11,17], but mostly affects cell number and size within the SAM. Concomitantly, SAM size is usually altered, with internal organization either affected severely, as occurs after downregulation or overexpression of B-type CDKs, or not affected, as is observed after overexpression of *CYCD3* and downregulation of *CDKA1* or *CYCD3* [8,11,15,18]. Studies also suggest that reduced division rates in the SAM exert a negative effect on proliferation outside of the meristem [18], and that root and shoot meristems may rely on different cell cycle regulators—for example, *CDKB2* seems to act on SAM but not RAM [8].

The most notable function of mitochondria is the generation of ATP through oxidative phosphorylation (OXPHOS), and consequently mitochondria are strongly associated with the production of reactive oxygen species (ROS) [19]. Furthermore, cell division is highly energetically demanding and related to increased oxygen consumption and concomitant ROS production [20]. Within the SAM and RAM, undisturbed mitochondria functioning is therefore especially valuable as proliferation is crucial for SAM and RAM functionality. Different cell division ratios are preserved in separate meristematic zones, which seems to be regulated by the cells redox status [7]. Importantly, the integration of hormone homeostasis, the expression of meristematic genes, and the cell division rate all seem to be orchestrated by redox homeostasis [20]. Remarkably, SAM is also unique in terms of being characterized by the presence of one large mitochondrion surrounding the nucleus, with only a few smaller mitochondria also present in the cell [21]. Changes in the architecture of mitochondria relate to cell cycle-dependent mixing of mitochondrial DNA, which, together with the requirements for proper delivery of ATP during cell proliferation, further emphasizes the uniqueness and vulnerability of meristematic cells.

One of the factors enabling the undisturbed maintenance of the SAM in specific growth conditions is AtFTSH4 [22], a mitochondrial metalloprotease with proteolytic and chaperone-like domains facing the intermembrane space (i-AAA) [23]. While comparable to *Arabidopsis* WT plants in standard growth conditions (long day photoperiod (LD), 22 °C) under short day photoperiod (SD), or LD with elevated temperature (30 °C), *ftsh4* mutants display striking phenotypic features in both vegetative and generative development. Loss-of-function *ftsh4* mutants form aberrant vegetative rosettes and shorter precociously terminating inflorescences, with an irregular pattern of side branches and flowers [22,24]. At*FTSH4* was found to be particularly important for SAM function around flowering time, protecting the stem cells against internal oxidative stress and maintaining the functionality of mitochondria [22]. Studies at the molecular level indicate the accumulation of oxidatively damaged proteins and other markers of oxidative stress in the loss-of-function *ftsh4* plants [22,24,25,26]. This accumulation is an intrinsic response to the disturbed functionality of OXPHOS complexes, ineffective removal of oxidized proteins arising from reduced ATP production, and altered mitochondrial dynamics causing restricted mitophagy [24]. Recently, it was also documented that AtFTSH4 can degrade oxidatively damaged proteins in isolated mitochondria [27]. In addition, mitochondria lacking AtFTSH4 have the enhanced capacity of preprotein import through TIM17:23-dependent pathway [28]. Furthermore, the loss of AtFTSH4 also influences processes outside mitochondria–ROS generated in the mitochondria of *ftsh4* plants interact with the phytohormone auxin and affect plant architecture [29], and it seems that AtFTSH4 regulate the expression of *WRKY* transcription factors that control salicylic acid synthesis and signaling in autophagy and senescence [30].

Taken together, there is a growing body of evidence that AtFTSH4 displays pleiotropic functions during plant growth and development primarily associated with internal oxidative stress [22]. In the present study, we focus on the role of AtFTSH4 in the premature termination of shoot and root meristems. We test the following hypotheses, that abrupt shoot and root growth cessation is associated with: (i) the disrupted expression of key SAM and RAM related genes; (ii) disrupted cell cycle progression; and (iii) the depletion of hormones; and that (iv) the termination of both SAM and RAM results from a similar underlying mechanism.

## 2. Results

We have previously shown that in standard growth conditions (long day photoperiod (LD), 22 °C) wild-type (WT) and *ftsh4-1* mutant plants are alike [22,24,25,26]. Therefore, in this study, we focus only on plants grown at 30 °C, the conditions which terminate meristem in *ftsh4-1* mutant but not in WT plants.

### 2.1. SAM of ftsh4-1 Mutants Display Reduced Proliferative Activity at 30 °C Prior to Flowering

**Figure 1:**
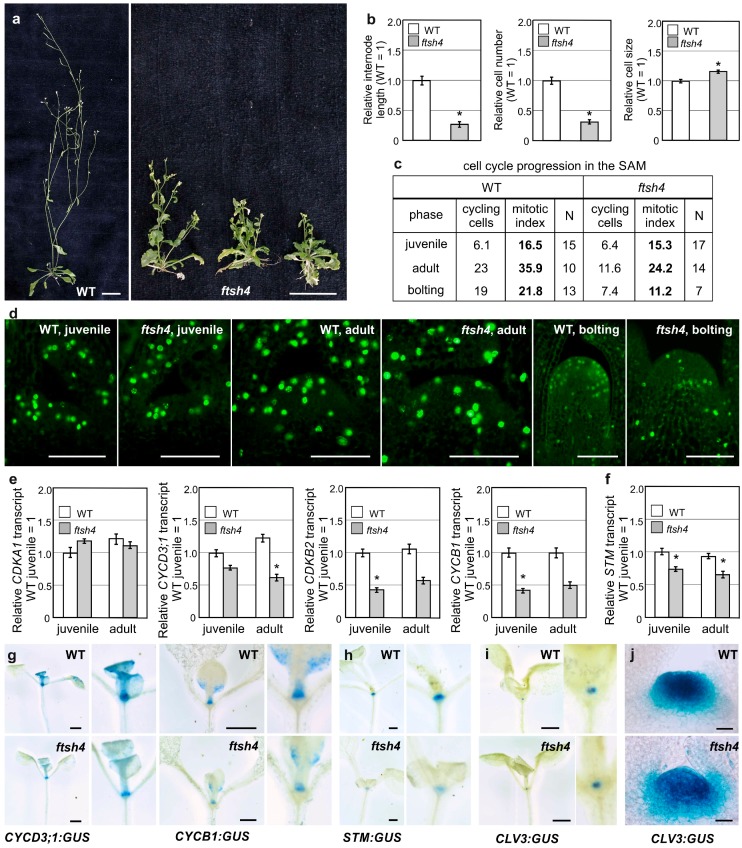

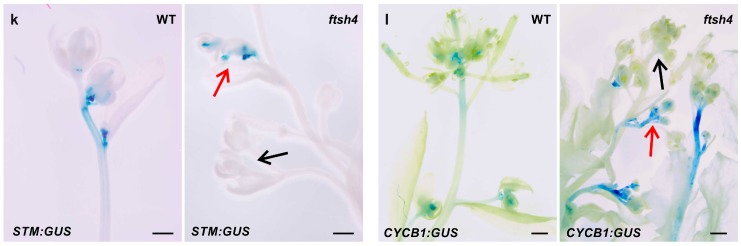
Impairment of proliferative activity and stem cell identity in shoots of *ftsh4-1* mutants grown at 30 °C. (**a**) Phenotypes of adult wild-type (WT) and *ftsh4-1* mutant plants grown under long day photoperiod (LD) conditions at 30 °C. The *ftsh4-1* mutants show a different degree of shortening of the main inflorescence stem and branching prior to growth cessation (note the drying rosette leaves). Scale bar: 20 mm; (**b**) lengths of the first internode and the number and size of cells in the first internode of WT and *ftsh4-1* mutant plants grown under LD conditions at 30 °C. The results are shown as relative to the WT samples (average values for WT plants are as follow: internode length 18.5 mm, cell number 54.3, elongation zone cells size 298.7 μm). Internode length and cell number strongly decreases, while cell size is less affected in the *ftsh4-1* mutants. Two biological replicates of the experiment were performed. The internode length and number of cells was measured in 10 plants of each genotype; average cell size was estimated from randomly measured 10 cells per internode. Mean values (±SE) are shown and significant differences between bars at *p* < 0.05 are denoted by asterisks; (**c**,**d**) comparison of S-phase progression (cell division) directly in the meristems of juvenile vegetative, adult vegetative and bolting wild-type and *ftsh4-1* mutants grown at 30 °C. The table shows the total number of cycling cells (i.e., in S-phase) and the mitotic index (percentage of cycling cells relative to the total number of SAM cells) in juvenile vegetative, adult vegetative and bolting wild-type and *ftsh4-1* mutant plants (**c**). In comparison to WT plants, a decrease in the number of S-phase cells (green signal) was detected only in adult and bolting *ftsh4-1* mutant plants. Scale bars: 50 μm (**d**); (**e**) comparison of At*CDKA1*, At*CYCD3;1*, At*CYCB1* and At*CDKB2;1* transcript levels analyzed in juvenile and adult vegetative shoot apices of WT and *ftsh4-1* mutant plants grown under LD conditions at 30 °C. Abundance of transcripts in each case is expressed relative to the juvenile WT tissue samples. Results from three experiments are shown; (**f**) the comparison of At*STM* transcript level analyzed in juvenile and adult vegetative shoot apices of WT and *ftsh4-1* mutant plants grown under LD conditions at 30 °C. Abundance of transcripts is expressed relative to the juvenile WT tissue samples. Results from three experiments are shown; (**g**,**h**) activity of the At*CYCD3;1* and At*CYCB1* (**g**) as well as At*STM* (**h**) promoters visualized by the activity of the GUS reporter protein (**blue**). Transgenic plants were grown under LD conditions at 30 °C and expression levels were analyzed during the juvenile vegetative stage of development of the WT (**upper panels**) and *ftsh4-1* mutants (**lower panels**). In each case, *ftsh4-1* mutants were characterized by weaker GUS activity. Scale bars: 3 mm; (**i**,**j**) activity of the At*CLV3* promoter visualized by the activity of the GUS reporter protein (**blue**). At 30 °C, in the juvenile SAM, GUS activity accumulates similarly in both genotypes (**i**), but in adult vegetative SAM (prepared with vibratome sections), *ftsh4-1* mutants are characterized by weaker and more diffusible GUS activity in comparison to the WT (**j**). Scale bars: 3 mm (**i**) and 20 μm (**j**); (**k**,**l**) activity of the At*STM* (**k**) and At*CYCB1* (**l**) promoters visualized by the activity of the GUS reporter protein (**blue**). Plants were grown under LD conditions at 30 °C and expression levels were analyzed during the generative stage of development. After transition to flowering, GUS activity in adult *ftsh4-1* mutant plants grown is variable (in terms of the strength of the signal) in the inflorescence apices (black arrows point exemplary shoot apices without GUS signal, red arrows—exemplary shoot apices with GUS signal). Scale bar: 15 mm.

In this study, we grew plants under LD conditions at 30 °C, which induces internal oxidative stress accumulation in *ftsh4-1* mutants and therefore the phenotype of precocious shoot and root termination [22]. To analyze the cellular basis of the *ftsh4-1* mutant’s main inflorescence stem shortening (Figure 1a), we analyzed the cell number and size in the first internode of flowering plants. In *ftsh4-1* mutants, the final cell number fell by over 50% when compared to WT plants, while cell size was negligibly affected (Figure 1b), indicating the impairment of proliferation in the SAM of mutants upon flowering. This result prompted us to monitor cell cycle progression directly in the SAM. For that purpose, we took the advantage of the Click-iT^®^ EdU Imaging Kit, which facilitates the visualization of cells in S phase. No difference was observed between juvenile WT and mutant plants, but in the adult vegetative and bolting stages of growth, the number of cycling cells in *ftsh4-1* plants gradually decreased compared to WT plants (Figure 1c,d). The decrease was related to both the total number and percentage (mitotic index) of S-phase cells within the SAM (Figure 1c).

As cell cycle cessation was evident even prior to flowering, we examined the expression of several genes related to G1-S and/or G2-M transitions in the shoot apices of juvenile and adult plants. Transcript levels for the major cell-cycle controlling kinase *CDKA1* were similar between the mutant and WT plants, but transcript levels for the kinase *CDKB2* and of two cyclins *CYCB1* and *CYCD3;1* (all three of which are documented to have an impact on meristem maintenance) were significantly reduced in mutants (Figure 1e). Importantly, the activity of the *pCYCB1* and *pCYCD3*;*1* promoters, driving the expression of the *GUS* reporter gene (*β-glucuronidase*; WT*/ftsh4-1*;*pCYCB1:GUS* and WT/*ftsh4-1*;*pCYCD3*;*1:GUS*), was detected in the shoot apical region, including the SAM and the youngest leaves, of juvenile vegetative plants, and was reduced in *ftsh4-1* mutants (Figure 1g). In line with these results, the expression of At*STM*, analyzed with use of qRT-PCR and a transgenic lines WT*/ftsh4-1;pSTM:GUS*, was reduced in the shoot apices of the juvenile *ftsh4-1* mutants in comparison to WT plants (Figure 1f,h). On the other hand, expression of the *CLV3* promoter (a stem cell marker) driving the expression of *GUS* gene (WT/*ftsh4-1;pCLV3:GUS*) in the SAM, was comparable in juvenile WT and mutant plants (Figure 1i) but became reduced and more diffuse in adult vegetative mutant plants (Figure 1j), confirming the strong impairment of SAM identity.

In the shoot apices of the flowering WT plants (grown at 30 °C), promoter activity was detected for cell cycle genes (*CYCB1*) in the SAM and the youngest flowers and for stem cell marker genes (*STM*) in the SAM as indicated by GUS reporter intensity and localization (Figure 1k,l), which is consistent with the literature data on plants grown under LD conditions at 22 °C (e.g., [31,32]). In the mutant grown at 30 °C, on the other hand, which formed short and irregularly branched stems, the analyzed genes were variably expressed within an inflorescence, with stronger, weaker, or even absent activity of the GUS in shoot apices. In addition, variation was present between plants, with the strength of the GUS signal not necessarily weaker in comparison to that in the WT plants (Figure 1k,l).

These findings indicate that the short and precociously terminated inflorescences in the *ftsh4-1* mutant are mostly the result of reduced cell number. The expression of the cell cycle related genes (*CYCB1*, *CYCD3*;*1*, and *CDKB2)* and meristematic gene *(STM)* are reduced in the juvenile phase, preceding the reduced proliferation activity and more deteriorated SAM identity in the adult vegetative plants (shown by reduced *CLV3* promoter activity). In addition to the arrest of cellular proliferation, after transition to flowering the *ftsh4-1* mutant plants are characterized by strongly dysregulated expression of cell cycle and meristem genes.

### 2.2. Cessation of Root Growth in ftsh4-1 Mutants Is Related to Termination of the Cell Cycle

**Figure 2:**
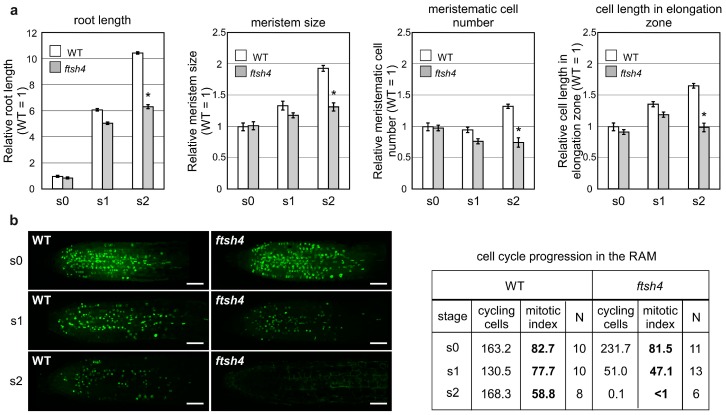

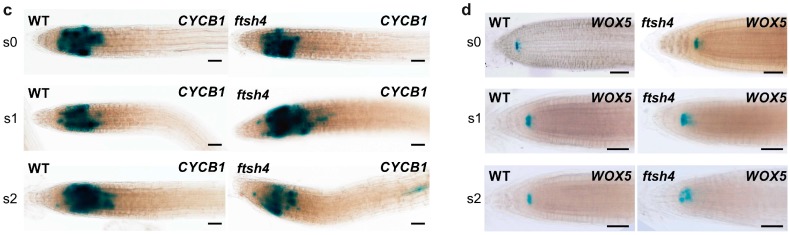
Impairment of proliferative activity and quiescence center (QC) cell identity in *ftsh4-1* mutant roots. (**a**) The length of the root and root apical meristem (RAM), the number of the RAM cells and the size of the cells in the elongation zone of wild-type (WT) and *ftsh4-1* mutant plants grown under long day photoperiod (LD) conditions at 30 °C. Seeds were germinated and grown for three days at 22 °C, and then transferred to 30 °C (S0) to continue growing for three days (S1) and six days (S2). Results are expressed relative to the WT S0 sample (average values for WT plants from S0 are as follow: root length 2.6 mm, meristem size 164 μm, meristematic cells number 14.4, cell length in elongation zone 127 μm). WT and mutant plants are comparable at S0 and S1 time-point, but then root and RAM size, RAM cell number and elongation cells size decreases in the *ftsh4-1* mutant S2 sample in comparison to the WT S2 sample. Two biological replicates of the experiment were performed. The root length, meristem length and meristematic cells number was measured in at least 10 plants of each genotypes; average size of cells in the elongation zone was estimated from randomly measured three cells per root. Mean values (±SE) are shown and significant differences between bars at *p* < 0.05 are denoted by asterisks; (**b**) comparison of S-phase progression (cell division) directly in the meristems of S0 (at the time of transfer), S1 and S2 RAM of wild-type and *ftsh4-1* mutants grown at 30 °C. A decreased in the number of S-phase cells (green signal) was detected in the S1 *ftsh4-1* mutant plants in comparison to WT plants. Scale bars: 40 μm. The table shows the total number of cycling cells (i.e., in S-phase) and the mitotic index (percentage of cycling cells relative to the total number of SAM cells) in S0, S1 and S2 wild-type and *ftsh4-1* mutant plants grown at 30 °C; (**c**,**d**) activity of the At*CYCB1* (**c**) and At*WOX5* (**d**) promoters visualized by the activity of the GUS reporter protein (blue). Transgenic plants were grown as described in (**a**) and expression levels were analyzed during the S0, S1 and S2 stages of development in the WT and *ftsh4-1* mutants. *ftsh4-1* mutants were characterized by comparable GUS activity for both promoters at S0 and S1 stages, only slightly weaker activity (not fully penetrant phenotype) in case of the At*CYCB1* promoter (**c**) and much weaker and diffusible in case of the At*WOX5* promoter (**d**) in the S2 stage. Scale bars: 40 μm.

Proliferative activity was also analyzed in the other apical meristem, the RAM, as *ftsh4-1* mutant plants are also characterized by prematurely ceased root growth when grown at 30 °C [22]. In the root growth experiments, seedlings were germinated and grown for three days at the optimal temperature of 22 °C (S0 plants) and then transferred to 30 °C for additional three (S1 plants) and six days (S2 plants) to analyze the cumulative effect of elevated temperature. Root lengths, RAM sizes and RAM cell numbers were comparable between the WT and *ftsh4-1* mutant plants at the moment of the transfer to 30 °C (S0) and after three days of growth (S1). Interestingly, after a further three days at the elevated temperature (S2), the roots and meristems of the mutants were shorter and the number of cells in the RAM less than the WT plants (Figure 2a). In addition, at the S2 stage, cells within the elongation zone of mutant plants were shorter in comparison to WT plants (Figure 2a).

Next, we analyzed the proliferative activity of the RAM (as indicated by the number of cells in the S phase) with the fluorescent EdU kit. In WT plants at both analyzed time-points (S1 and S2), the proliferation was equally distributed throughout the RAM (Figure 2b) and maintained at a relatively high level as shown by the number and percentage (mitotic index) of cycling cells (77.7% in S1 and 88% in S2). On the contrary, the number and percentage of cycling cells were strongly reduced in the mutant plants at S1, with the proliferative activity concentrated closer to the root tip. Concomitantly, after subsequent three days at 30 °C (S2), cell division in the mutant plants had almost completely ceased (Figure 2b). Interestingly, the expression of p*CYCB1*:*GUS* was comparable between WT and *ftsh4-1* mutant plants in the S1 stage and only slightly weakened at S2 in some mutants (Figure 2c). On the other hand, the expression of the QC marker p*WOX5*:*GUS* was comparable between both genotypes at the S1 stage, but was noticeably weaker, more diffuse, or even almost completely gone in *ftsh4-1* mutant plants at S2 compared to the WT (Figure 2d).

In summary, after three days at 30 °C, root growth in the *ftsh4-1* mutant ceased, which is consistent with the observed limited cell cycle progression within the RAM, despite the maintenance of *CYCB1*:*GUS* expression. In addition, *WOX5* expression weakened after six days, but not three days, of growth at 30 °C.

### 2.3. Hormonal Insensitivity of the SAM and RAM of the ftsh4-1 Mutant

To test whether impaired SAM and RAM maintenance in plants lacking the At*FTSH4* gene is attributable to the deficiency of a particular hormone, we analyzed the impact of the exogenous application of hormones on shoot and root growth in plants grown at 30 °C.

**Figure 3:**
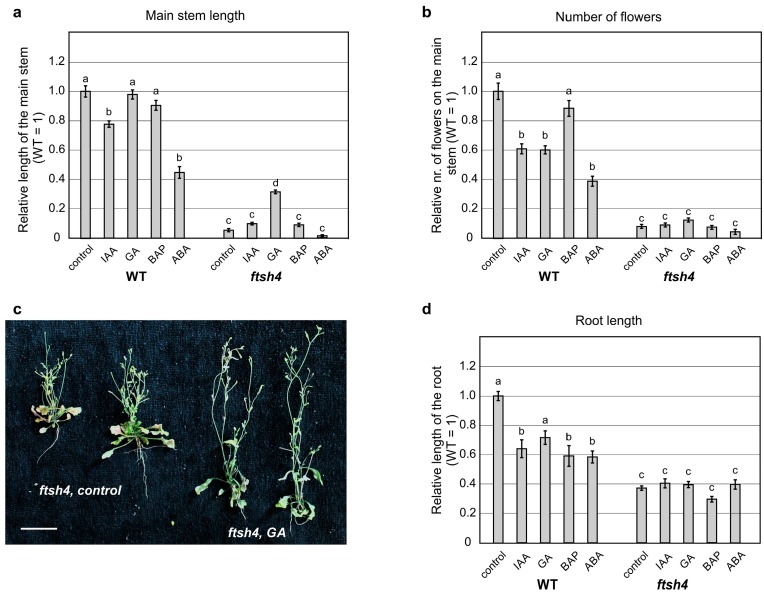
Lack of responsiveness to exogenous hormones in the shoots and roots of the *ftsh4-1* mutants grown at 30 °C. (**a**,**b**) Height of the main inflorescence (**a**) and the number of flowers on the main inflorescence (**b**) of wild-type (WT) and *ftsh4-1* mutant plants grown under long day photoperiod (LD) conditions at 30 °C. The results are expressed relative to the WT control sample (plants without exogenous hormone application). Average values for WT control plants are as follow: main stem length 35.6 mm, number of flowers 21. The main inflorescence height of the *ftsh4-1* mutants increases after gibberellic acid (GA_3_) application (**a**), but the number of flowers is not significantly changed (**b**). Mean values (±SE) are shown; (**c**) the phenotype of adult (when rosette leaves are drying) *ftsh4-1* mutant plants grown under LD conditions at 30 °C. *ftsh4-1* mutants without exogenous hormone application are shown on the left, and those after gibberellic acid application are shown on the right. Scale bar: 20 mm; (**d**) the length of the main root of WT and *ftsh4-1* mutant plants, grown under LD conditions at 30 °C. Seeds were germinated and grown for three days at 22 °C, transferred to 30 °C to continue the growth for six days and then analyzed. The results are shown relative to the WT control sample (without exogenous hormone application). Mean values (±SE) are shown and significant differences in the WT and *ftsh4-1* mutant plants after various hormones application in comparison to their control plants (WT or *ftsh4-1*), at *p* < 0.05, are indicated with different letters: a and b between treated and not-treated WT plants; c and d between treated and not-treated *ftsh4-1* plants.

When shoot growth was analyzed after treatment with auxin and cytokinin, no significant change in the WT plants or the mutant’s short inflorescence was observed (Figure 3a and Figure S1), while abscisic acid significantly reduced the inflorescence of both the mutant and WT plants (Figure 3a and Figure S1). When gibberellic acid (GA_3_) was applied, no change to WT plant’s height was observed. Interestingly, the length of inflorescence in the *ftsh4-1* mutants increased, without significant increase in flower number (Figure 3a–c and Figure S1). In addition, the application of GA_3_ caused precocious flowering of both WT plants and *ftsh4-1* mutants, which occurred around six days earlier than in control plants (Figure S2).

To test root growth, the same hormones mentioned above were applied after seed germination at 22 °C for three days and subsequent transfer to 30 °C. When growing at 30 °C, all hormones reduced the length of the main root in the WT plants (Figure 3d and Figure S1). In contrast, *ftsh4-1* roots did not significantly respond at all to any of the hormones (Figure 3d and Figure S1).

In summary, the size of the main inflorescence increased slightly in the *ftsh4-1* mutant after GA_3_ application, with no increase in the organogenic activity of the SAM, while the length of the main root did not increase after application of the hormones.

## 3. Discussion

**Figure 4:**
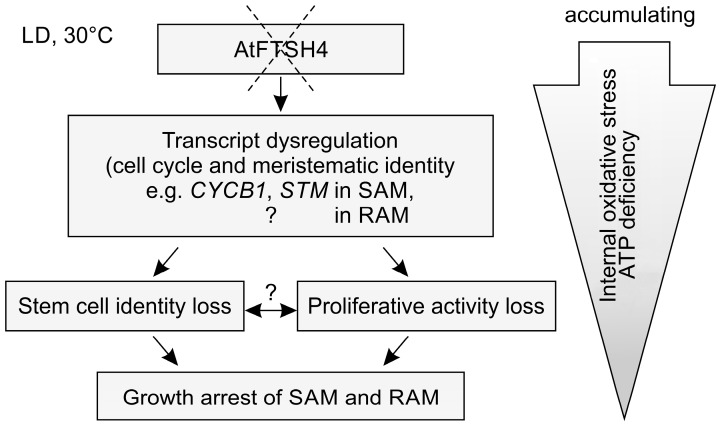
Developmental impairment of meristem proliferation and stem cell identity in plants lacking the mitochondrial protease AtFTSH4. In stress-inducing conditions of mildly elevated temperature of 30 °C, absence of AtFTSH4 in mitochondria (shown as dashed X) results in progressive accumulation of internal oxidative stress (ROS, carbonylated proteins, *AOX* transcripts) with time/plant age and ATP deficiency [22,24,25]. Stem cells within the meristems lose their meristematic characteristics and ability to proliferate. In the shoot apical meristem (SAM), this relates to the transcript dysregulation of cell cycle-related genes and those sustaining meristematic identity. Ultimately, growth of the SAM and RAM in the *ftsh4-1* mutant plants is precociously arrested. Question marks refer to probable, but not yet proven experimentally correlations.

AtFTSH4 protease is essential for the maintenance of healthy mitochondria function and to counteract internal oxidative stress accumulation in plants growing under stress-inducing conditions. It therefore plays an important role in the functioning of the whole plant. As previously reported, AtFTSH4 is particularly important for the maintenance of the meristematic identity [22]. This study highlights that the phenotype characterized by precocious cessation of shoot and root growth arises from accumulating defects in various processes occurring at the tissue and molecular levels, including reduced proliferative activity in the shoot and root apical meristems (SAM and RAM, respectively) and dysregulated expression in the SAM of meristematic genes and those related to cell cycle control (Figure 4).

Here, we showed that proliferation activity decreases with time/age in both meristems of mutant plants. In the SAM, the expression of *CYCB1*, *CYCD3;1* and *CDKB2* decreases in the *ftsh4-1* juvenile plants when compared to the wild-type (WT). At the same time point, cell cycle progression, visualized directly in the SAM, was not yet affected. Concomitantly, the proliferation rate gradually declines in the adult vegetative mutants, a phenomenon that continues after bolting. In addition, the expression of stem cell related genes, *STM* and *CLV3*, is reduced in the juvenile and adult stages, respectively. Similar to the SAM, growth cessation in the RAM was correlated to the gradually reduced proliferation. In this case, the termination of cell division was not preceded by the reduced level of *CYCB1* expression. The reduced expression of *CYCB1*, and also *WOX5* (which is a positive regulator of stem cell maintenance expressed in the quiescent center, QC), followed the loss of RAM proliferative activity and thus ongoing differentiation. We cannot, however, rule out the possibility that in the root a disturbance in the expression of genes other than *CYCB1* could drive the cessation of proliferation, as literature data suggest that shoots and roots rely on different cell cycle genes to integrate proliferation with meristem organization [8]. Moreover, most mitochondrial mutants affecting OXPHOS system are characterized by the phenotype of short roots and/or shoots, and that decreased growth rates can be linked to the reduced proliferation [33,34,35]. Thus, at least some aspects of SAM and RAM termination, as direct responses to accumulation of reactive oxygen species (ROS) and/or ATP deficiency, could be similar. It would be interesting in the future to compare in more detail the mechanisms behind SAM and RAM termination to decipher differences and similarities, also between the two meristems, across different mitochondria mutants affecting OXPHOS system.

These findings agree with our previous results showing that internal oxidative stress (ROS, carbonylated proteins, and *AOX* transcripts) and concomitant mitochondria dysfunction accumulate progressively [22]. The oxidative environment of the adult vegetative SAM thus causes a gradual loss of the SAM identity and proliferative ability. Nevertheless, upon transition to flowering, proliferative activity is preserved, though limited, and the stem cell character of the cells in the SAM is maintained sufficiently for generative development to progress. However, the inflorescence stems terminate precociously. In such plants, the expression of *CYCB1* and *STM* was not uniform, suggesting their significant dysregulation in mutant plants after or during flowering. The observed differential expression levels probably result from a varying degree of defects caused by accumulating internal oxidative stress, and reveals inherent variability in the fitness of individual meristems. From a whole plant perspective, such differences reflect a strong dysregulation of processes at multiple levels of organization.

Cell cycle and developmental programs need to be coordinated by hormonal signals, and hormonal outputs are coordinated by the cell cycle machinery [2,11]. Disrupted *CDKB2* function, for example, causes an elevation in auxin levels, a reduction in bioactive cytokinins, and an inability to properly interpret hormonal stimuli in a developmental output [8]; *CYCD3;1* is induced, among other stimuli, by sucrose and cytokinin [9,36,37]. Interestingly, two genes crucial for SAM maintenance, *STM* and *WUS,* are related to cytokinin [38,39], and maintenance of low mitotic activity in RAM QC cells was shown to depend on their highly oxidized status and proper auxin levels (which are dependent on redox homeostasis) [7]. The loss of apical dominance of the main inflorescence stem in *ftsh4* mutants was reported to be complemented with external auxin application [29]. However, the phenotype of the *ftsh4-1* mutant grown at 30 °C is rather pleiotropic and not purely the result of disturbed levels of or responses to cytokinin, auxin, or any other hormone. In our studies, only the application of gibberellic acid (GA_3_) resulted in minor complementation of the shoot growth defects exhibited by *ftsh4-1* mutant plants grown under stress-inducing conditions. However, this might be simply the result of reduced accumulation of internal oxidative stress, and therefore less impact on SAM function, as these plants flowered earlier than the control *ftsh4-1* mutants. In addition, the roots of the *ftsh4-1* mutant were generally less responsive than WT to all exogenous hormones. These results support the assumption that the developmental defects observed in the *ftsh4-1* mutant induced by the accumulation of internal oxidative stress are multicomponent and cannot be explained by changes in any single process. At this stage, it is impossible to assess whether the driving force behind the impairment of proliferation is a disturbance in hormonal homeostasis interfering with cycling, or rather cell cycle related genes influencing hormonal homeostasis.

Our results show that growth arrest in the *ftsh4-1* mutant is caused not only by a loss of stem cell identity, but importantly, dysregulation of the cell cycle. SAM and RAM termination in plants lacking the mitochondrial protease AtFTSH4 is an outcome of progressively declining proliferation rates and stem cell maintenance due to progressive internal oxidative stress accumulation associated with ongoing mitochondria dysfunction. In a final attempt to survive, *ftsh4-1* mutants undergo transition to flowering, but form only defective inflorescences that fail to produce seeds and precociously cease development.

## 4. Materials and Methods

### 4.1. Plant Material and Growth Conditions

*Arabidopsis thaliana* (L.) Heynh. Columbia-0 (Col-0) was used as the wild type (WT) reference. The transgenic lines *ftsh4-1* (SALK_035107/TAIR) line was already previously characterized [25], and was originally obtained from the Salk Institute. Other transgenic lines used in this study were obtained: *pSTM:GUS* from Prof. W. Werr (University of Cologne, Cologne, Germany), *pCLV3:GUS* from Prof. T. Laux (University of Freiburg, Freiburg, Germany), *pCYCB1:GUS* from Prof. N. Dengler (University of Toronto, Toronto, ON, Canada), and *pCYCD3;1:GUS* from Prof. J. Murray (Cardiff School of Biosciences, Cardiff, UK). Plants were grown in a 16 h light/8 h dark (long day, LD) photoperiod at 22 °C and 30 °C. The transgenic lines used in that study were created by means of crossing the *ftsh4-1* mutant to the respective GUS reporter line. Concomitantly, double homozygous plants (homozygous for the GUS reporter and homozygous for wild-type or mutated allele of the At*FTSH4* gene) were selected. Hormones were exogenously applied at following concentrations: 100 μM indole-3-acetic acid (IAA), 100 μM gibberellic acid (GA_3_), 1 μM 6-benzylaminopurine (BAP), and 10 μM abscisic acid (ABA) for the shoot activity; and 0.5 μM IAA, 50 μM GA, 0.5 μM BAP, and 10 μM ABA for root activity.

### 4.2. Histological Analyses

*GUS* gene activity, under the control of the At*STM*, At*CYCB1*, At*CYCD3;1*, and p*CLV3* promoters, was analyzed in *Arabidopsis* plants grown in LD at 30 °C at various developmental stages. Tissues were prefixed in ice-cold 90% (*v*/*v*) acetone, rinsed with 50 mM sodium phosphate buffer (pH 7.2) and then stained with 2 mM X-gluc (5-bromo-4-chloro-3-indolyl β-d-glucuronide cyclohexamine; Duchefa Biochemie, Haarlem, The Netherlands) for 3, 16, 16, and 7 h, respectively, in the dark, at 37 °C. Concomitantly, plants were treated with increasing ethanol concentrations, and fixed with the 50% ethanol-3.7% formaldehyde-5% acetic acid (FAA) solution or stored in ethanol. Longitudinal sections of the SAM (25 µm thick) were prepared on a vibratome (Leica VT 1200S; Leica Instruments GmbH, Wetzlar, Germany) using both juvenile and adult plants. Images were obtained using a stereomicroscope or light microscope (see Section 4.5). To observe cell number in the internodes, tissues from the *ftsh4-1* mutant and WT were dissected from fully adult flowering plants (around 20 plants for each genotype), fixed in FAA solution, hydrated in decreasing ethanol concentrations, and digested with 10% KOH for three days in 37 °C. The tissues were then rinsed extensively in water, dehydrated in increasing ethanol concentrations, and stained with nigrosine solution. The plant material was then documented using a stereomicroscope and an epi-fluorescence microscope (see Section 4.5).

### 4.3. Real-Time PCR

The shoot apices (SAM and youngest primordia) were hand dissected from at least 10 individual plants of each genotype, at the same time (9:00 a.m.), at the stage when the third or fifth leaf was visible (for juvenile and adult stages, respectively). Real-time PCR analyses were performed using a LightCycler480 (Roche, Penzberg, Germany) and Real-Time 2× PCR Master Mix SYBR version B (A&A Biotechnology, Gdynia, Poland) with a final primer concentration of 0.5 μM. Material from a pool of WT plants served as the calibrator, using the *PP2AA3* gene (At1g13320) as a reference. Amplification conditions comprised denaturation at 95 °C for 1 min followed by 45 cycles of amplification at 95 °C for 10 s, 55–65 °C (according to primer-specific annealing temperatures) for 10 s, and 72 °C for 20 s with single data acquisition, followed by cooling to 40 °C for 30 s. Melting curve analysis was performed as to test the specificity of the amplification products was. Real-time PCR analyses were performed on at least three independent biological replicates. The primers sequences are available upon request.

### 4.4. SAM in Vivo Fluorescent Analyses

Proliferation rates (S phase progression) were measured using a fluorescent Click-iT^®^ EdU Imaging Kit (Thermo Fisher Scientific, Waltham, MA, USA) directly in the SAM and RAM. The nucleoside analog of thymidine, EdU (5-ethynyl-2′-deoxyuridine), was applied to the plant tissues for 1 h through the hypocotyl for the SAM and by submerging the roots in a solution in the case of RAM. EdU incorporated into DNA during DNA synthesis was labelled with Alexa Flour 488 according to the manufacturer’s protocol. As a negative control, plants without the application of the Click-iT^®^ reaction cocktail were analyzed. For SAM analysis, EdU was applied to juvenile vegetative, adult vegetative, and bolting (with the inflorescence stem showing early signs of growth) WT and *ftsh4-1* plants grown under LD at 30 °C. To analyze RAM, EdU was applied to the roots of six- and nine-day-old plants, which were first grown for three days at 22 °C and then transferred to 30 °C for three and six days to bypass defects related to germination. A confocal laser scanning microscope (CLSM, see Section 4.5) was used to detect the fluorescent signal of Alexa Flour 488 in at least 10 longitudinal median sections of individual SAM or whole mount roots. The mitotic index was calculated as the percentage of all cycling cells relative to the total number of the cells in the meristem.

### 4.5. Microscopy

The plant material and prepared slides were, after above-mentioned techniques, photographed by the following equipment: (a) a digital camera; (b) a stereomicroscope with a digital camera and DLTCam software (Delta Optical, Nowe Osiny, Poland); (c) an epi-fluorescence BX60 microscope with bright-field optics equipped with a digital camera DP73 and cellSens Entry software (Olympus Optical, Tokyo, Japan); and (d) an inverted CLSM (Fluo View100; Olympus Optical, Tokyo, Japan). Alexa Flour 488 was analyzed in CLSM using the excitation and emission wavelengths of 495 and 519 nm, respectively.

### 4.6. Statistical Analyses

The data did not have a normal distribution (Shapiro-Wilk test, α = 0.05); thus, the significance of differences between independent groups was checked using Mann-Whitney *U* test. Statistical analyses were performed with Statistica 13 software (StatSoft; North Melbourne, Victoria, Australia).

## Supplementary Materials

Supplementary materials can be found at http://www.mdpi.com/1422-0067/19/3/853/s1.
