# Supplementary material for: Impairment of Meristem Proliferation in Plants Lacking the Mitochondrial Protease AtFTSH4
Source: Int J Mol Sci. 2018 Mar 14;19(3):853. doi: 10.3390/ijms19030853 (PMC5877714; doi:10.3390/ijms19030853)
Supplement: Supplementary file 1 [file ijms-19-00853-s001.pdf]

A

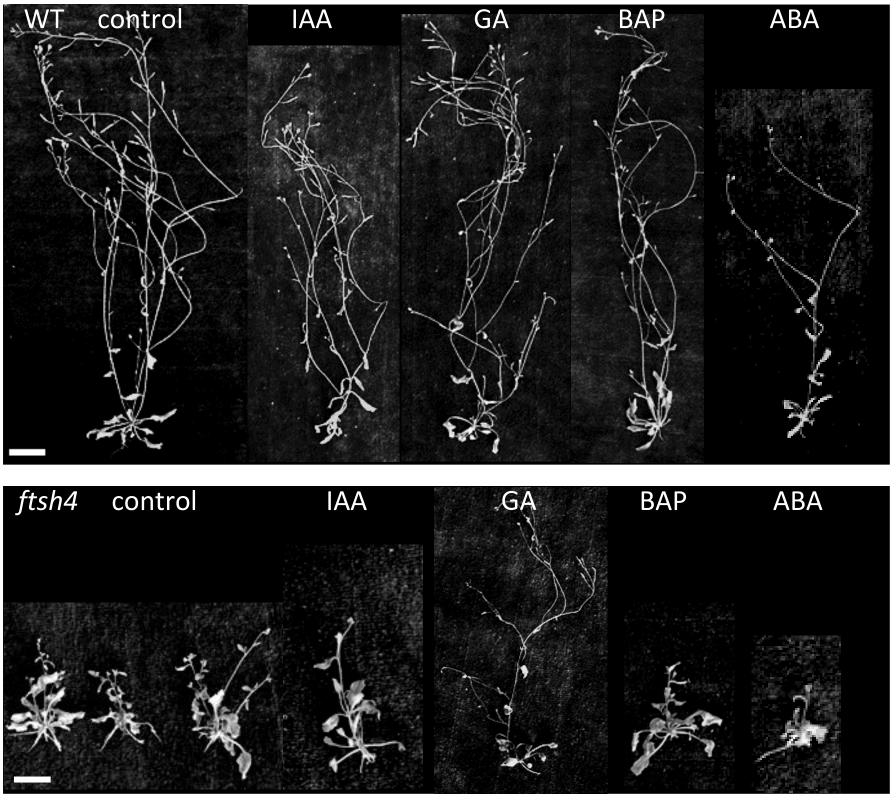

B

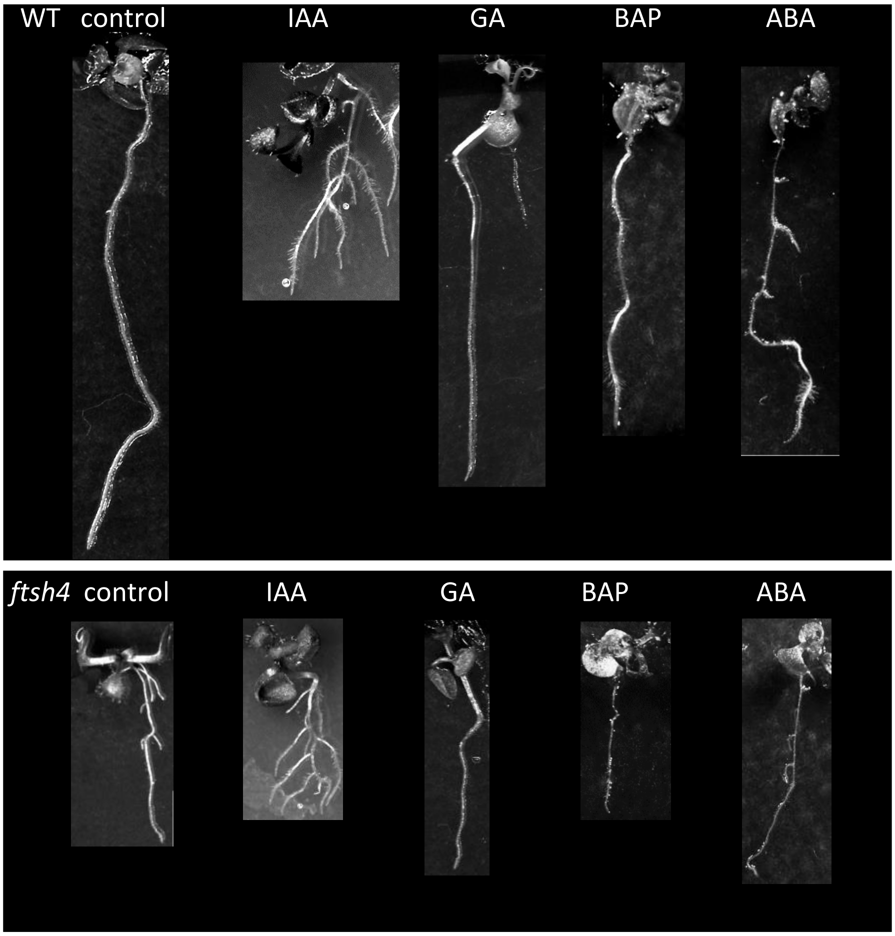

Figure S1. Response to exogenous hormones of the shoots and roots of the WT and *ftsh4-1* mutant plants grown at 30 °C.

The phenotype of WT and *ftsh4-1* plants without (control) and after application of various hormones (IAA, GA3, BAP, ABA). The fully adult, flowering plants ar shown in (a), and roots are shown in (b). WT plants are shown in upper panels and *ftsh4-1* plants are shown in lower panels.

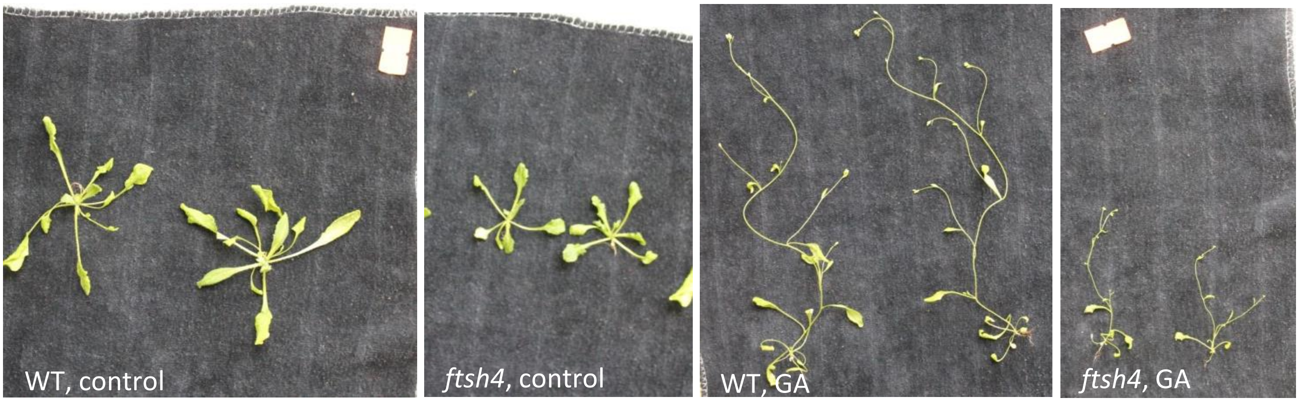

Figure S2. Phenotype of early flowering after gibberellic acid application in plants grown at 30 °C.

Control *ftsh4-1* plants after gibberellic acid application exhibit early flowering (6 days earlier than control) control *ftsh4-1* plants (without exogenous hormone applications) are still in the adult vegetative stage. *ftsh4-1* control mutant plants are shown on left, and mutant plants following gibberellic acid application are shown on right.
